# Extracellular RNAs as potential biomarkers for cancer

**DOI:** 10.20517/2394-4722.2020.71

**Published:** 2020-09-17

**Authors:** Christine Happel, Aniruddha Ganguly, Danilo A. Tagle

**Affiliations:** 1 National Center for Advancing Translational Sciences, National Institutes of Health, Bethesda, MD 20892, USA.; 2 Cancer Diagnosis Program, Division of Cancer Treatment and Diagnosis, National Cancer Institute at the National Institutes of Health, Bethesda, MD 20892, USA.

**Keywords:** Extracellular vesicles, exosomes, extracellular RNA, cancer, biomarker, liquid biopsy

## Abstract

The discovery that all cells secrete extracellular vesicles (EVs) to shuttle proteins and nucleic acids to recipient cells suggested they play an important role in intercellular communication. EVs are widely distributed in many body fluids, including blood, cerebrospinal fluid, urine and saliva. Exosomes are nano-sized EVs of endosomal origin that regulate many pathophysiological processes including immune responses, inflammation, tumour growth, and infection. Healthy individuals release exosomes with a cargo of different RNA, DNA, and protein contents into the circulation, which can be measured non-invasively as biomarkers of healthy and diseased states. Cancer-derived exosomes carry a unique set of DNA, RNA, protein and lipid reflecting the stage of tumour progression, and may serve as diagnostic and prognostic biomarkers for various cancers. However, many gaps in knowledge and technical challenges in EVs and extracellular RNA (exRNA) biology, such as mechanisms of EV biogenesis and uptake, exRNA cargo selection, and exRNA detection remain. The NIH Common Fund-supported exRNA Communication Consortium was launched in 2013 to address major scientific challenges in this field. This review focuses on scientific highlights in biomarker discovery of exosome-based exRNA in cancer and its possible clinical application as cancer biomarkers.

## INTRODUCTION

Once thought to exist only within cells, RNA is now known to play a role in a variety of complex cellular functions. Recent research has shown that RNA can be exported from cells and plays a role in the molecular mechanisms of cell-to-cell communication^[1,2]^. This paradigm-shifting observation launched the field of extracellular RNA (exRNA) biology and represents a fundamental change in our understanding of RNA in cell biology.

Extracellular RNA acts as a signalling molecule, traveling though body fluids carrying information from cell to cell. Types of exRNA include both longer messenger RNA (mRNA) and long non-coding RNA (lncRNA), as well as various types of small non-coding RNAs (ncRNAs). Non-coding RNAs can generally be broken down into two groups, regulatory ncRNAs and housekeeping ncRNAs, as outlined in Table 1. Regulatory ncRNAs include lncRNA, microRNA (miRNA), piwi-interacting RNA (piRNA), small interfering RNA (siRNA), tRNA-derived fragments and Y RNA. Regulatory small ncRNAs have emerged as vital players in various biological processes. They are known primarily for their role as regulators of gene expression at the post-transcriptional level; however, they have a wide range of functions. Further information on individual ncRNAs can be found in the review articles cited in Table 1. Housekeeping ncRNAs include ribosomal RNA (rRNA), transfer RNA (tRNA), small nuclear RNA (snRNA), and small nucleolar RNA (snoRNA). Housekeeping ncRNAs are highly abundant and are essential for cellular activities such as the translation of RNA into proteins, and transcriptional splicing. The term exRNA includes many types of RNA. Small non-coding exRNAs are often the focus of studies due to their abundance, ease of detection, and regulatory function. MiRNA are of particular interest due to their role in post-transcriptional regulation of gene expression. Changes in miRNA expression are associated with various pathological conditions and dysregulation of miRNA expression is a hallmark of human cancer^[3]^.

**Figure 1.**
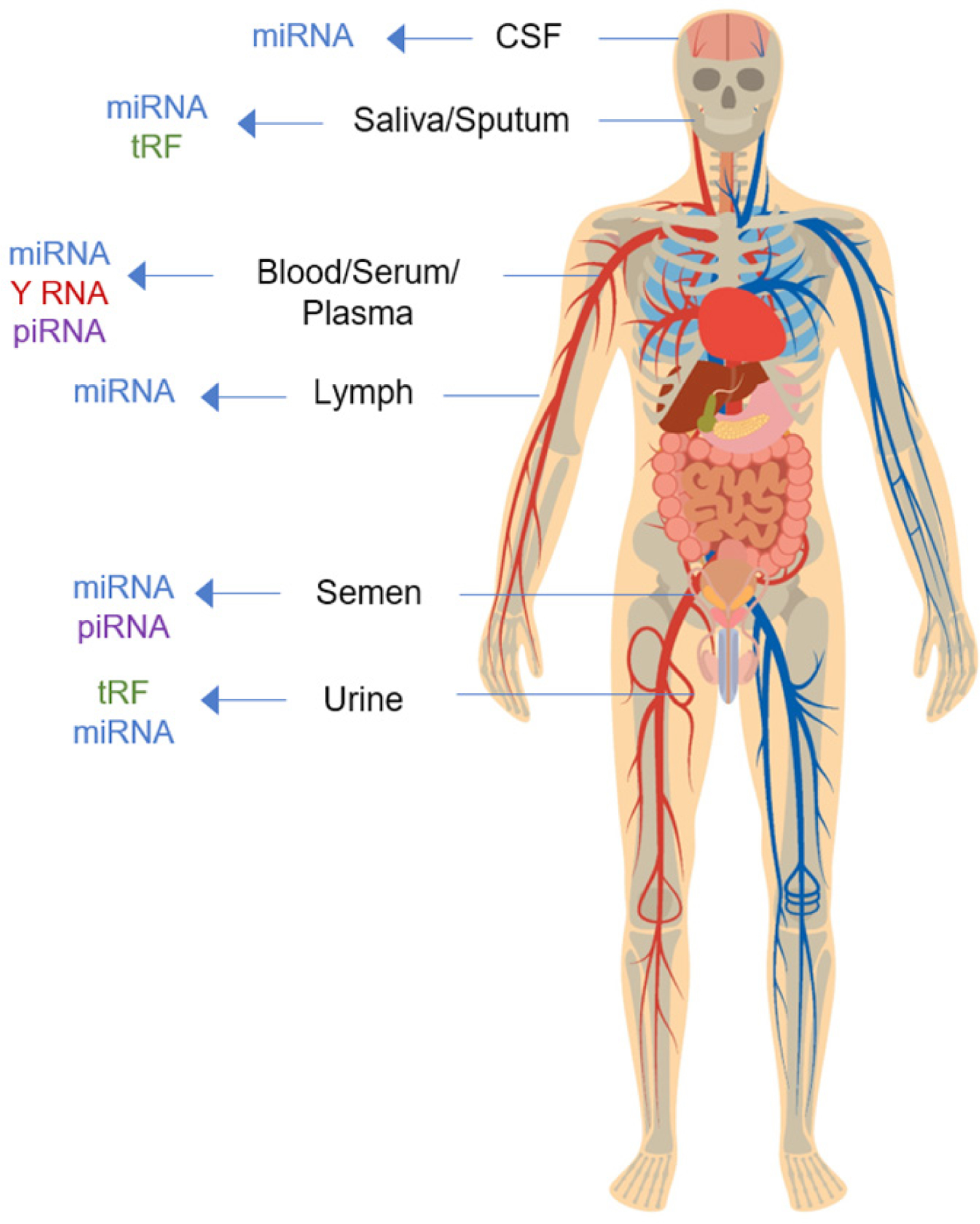
A schematic diagram showing exRNA types predominantly found in a representative set of human biofluids. miRNA: microRNA; piRNA: piwi-interacting RNA; tRF: tRNA-derived RNA fragments

Extracellular RNA is secreted by all cell types and can be found in a variety of biofluids including plasma, serum, breast milk, saliva, cerebrospinal fluid (CSF), bile, semen, and urine^[4–7]^. While many ncRNAs are found in human biofluids, miRNA, piRNA, snoRNA, tRNA-derived RNA fragments (tRF), and Y RNA represent the most prominent types of exRNA found within various human biofluids (Figure 1 and Table 1, asterisks)^[4,8]^. Carriers of exRNA include extracellular vesicles (EVs), ribonucleoprotein complexes (RNPs), and lipoprotein complexes (LPPs). ExRNAs are either encased within extracellular vesicles, or, are tightly associated with proteins to avoid degradation by RNAses. ExRNAs, in extracellular vesicles and/or associated with protein complexes, can then be transferred from donor cells to recipient cells, where they can elicit functional responses and regulate a number of biological processes^[9,10]^.

**Figure 2.**
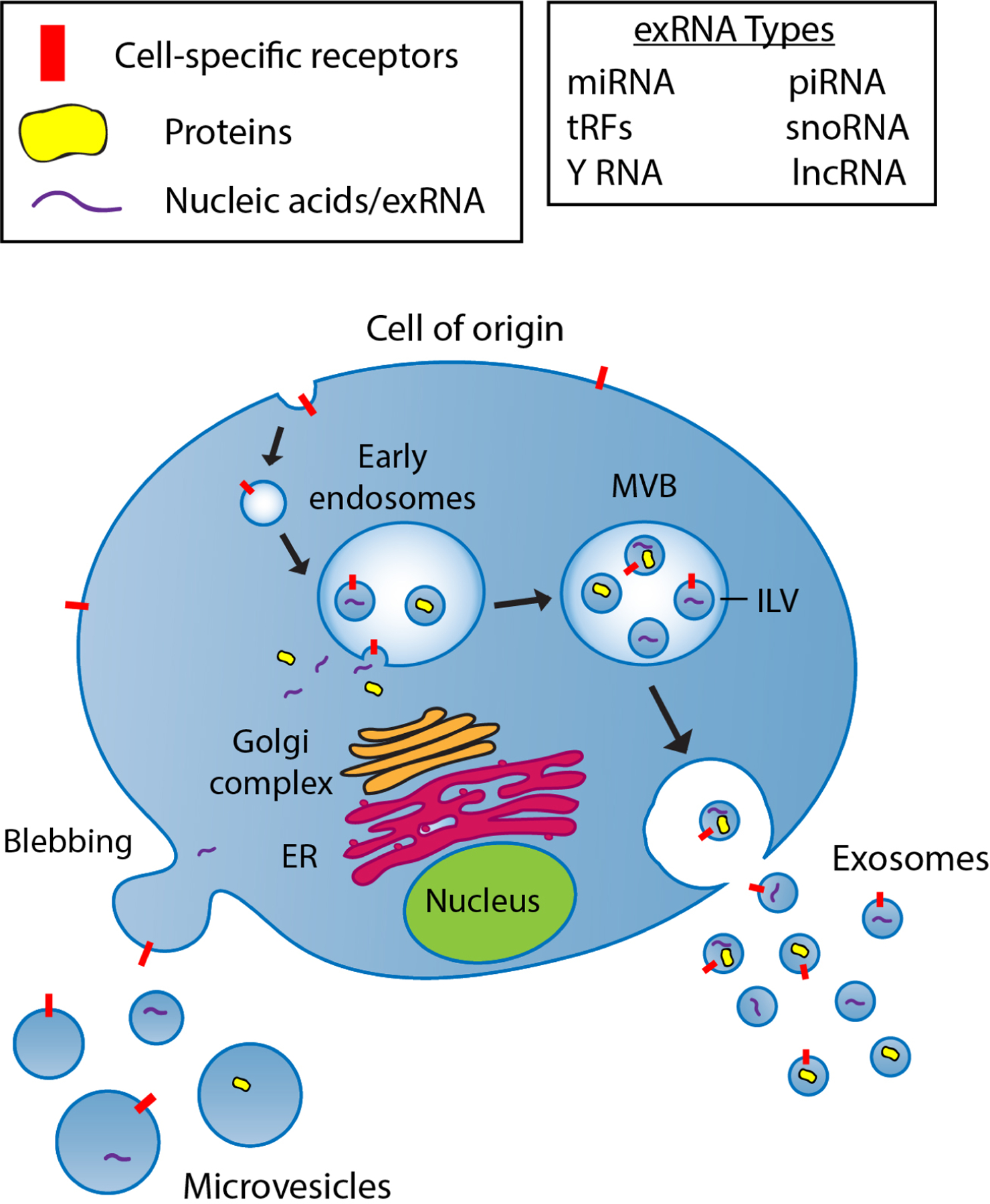
A schematic diagram showing the biogenesis pathway of microvesicles and exosomes. Microvesicles are formed by direct budding from the plasma membrane and are capable of encapsulating multiple forms of molecular cargo including proteins and nucleic acids. The biogenesis of exosomes begins with internalization of the cell membrane leading to the formation of early endosomes. Intraluminal vesicles (ILVs) are formed by the inward invagination of endosomal membranes, resulting in the formation of multivesicular bodies (MVBs). During this process, cytosolic constituents, including nucleic acids and proteins, can be sorted into ILVs. Upon fusion of MVBs with the plasma membrane, ILVs are released as exosomes into the extracellular milieu. Exosomes can include many different types of exRNA as listed in Table 1. ER: endoplasmic reticulum; MLV: multivesicular body; ILV: intraluminal vesicle; miRNA: microRNA; piRNA: piwi-interacting RNA; tRF: tRNA-derived RNA fragments; snoRNA: small nucleolar RNA; lncRNA: long non-coding RNA

EVs, released by virtually all cell types, are small membrane-enclosed carriers of bioactive proteins, lipids, and nucleic acids (including exRNAs)^[11]^. Cells release a variety of EVs to transfer biological cargo to local and distant recipient cells within the body to facilitate intercellular communication. The term extracellular vesicles is broadly used for particles released from the cell that are delineated by a lipid bilayer, however, there are multiple EVs subtypes which can be differentiated based on their size, biogenesis, release pathways, cargo, and function^[12]^. The main EV subpopulations include microvesicles (MVs), and exosomes. MVs are approximately 100–1000 nm in size and are derived from outward blebbing of the plasma membrane. Exosomes are approximately 30–100 nm vesicles of endosomal origin^[13]^. The biogenesis of exosomes begins with the formation of early endosomes by inward budding of the cell membrane, followed by second inward budding of the endosomal membrane creating intraluminal vesicles (ILVs) and the larger multivesicular bodies (MVBs). Fusion of the MVBs with the plasma membrane release ILVs as exosomes into the extracellular milieu. Cytosolic constituents such as proteins and nucleic acids can be sorted into both types of EVs as part of their respective biogenesis pathways [Figure 2].

Non-vesicle associated carriers include ribonucleoprotein (RNP) and lipoprotein (LPP) complexes. These non-membrane bound exRNA carriers have been shown to be present in human plasma and serum^[14,15]^. The LPP family of complexes are classically regarded as carriers of lipids and can be further broken down into high-density lipoproteins (HDLs), low-density lipoproteins (LDLs), very LDLs (VLDLs), and chylomicrons based on their mass density. Recent studies have revealed that lipoproteins, such as HDLs and LDLs, can transport miRNAs and deliver them to recipient cells where they carry out their functional roles^[16,17]^. One of the main interests in exRNA research is focused on their ability to mediate intercellular communication and act as signalling molecules in normal cell homeostasis, or as a consequence of pathological development. The exRNAs demonstrated potential as cancer biomarkers due to their function. There are published reports to support the use of exRNA for both cancer diagnosis and prognosis^[18]^.

This article focuses on exosome-derived exRNAs obtained non-invasively from liquid biopsy as potential biomarkers for the early detection and monitoring of cancers. Developing biomarkers based on exRNA is relevant and important in the context of precision cancer therapy, since this approach will result in non-invasive procedures using body fluids as test samples, and essentially eliminate unnecessary repeat biopsies for diagnosis and monitoring effectiveness of a therapy^[19]^. ExRNAs are now being evaluated as biomarkers in a variety of cancers and this review provides an understanding of the present status of exosome/exRNA-based cancer biomarker research, acknowledges challenges, and addresses the need to identify, develop, and validate clinically relevant exosomal exRNAs as cancer biomarkers.

## LIQUID BIOPSY

Liquid biopsy is a term generally used to describe the collection of a body fluid to test for diagnostic information that will guide patient management. Various biological fluids can be used for liquid biopsies, but blood is one of the most accessible fluids along with urine and saliva^[20]^. The ultimate goal of liquid biopsies in cancer patients is to be informative about the underlying tumour biology and establish biomarker clinical utility with clear prognostic value. Non-invasive measurement of cancer biomarkers using liquid biopsy allows for patient stratification, screening, monitoring treatment response, and detecting minimal residual disease following therapy/surgery and recurrence. The emergence of sensitive nucleic acid and protein biomarkers detection technologies have enabled the development of liquid biopsies with clinical applications in oncology. Currently, tumour biopsy is the preferred diagnostic tool available to clinicians to detect and monitor treatment for cancer. Since many tissues are difficult or impossible to biopsy or resect, and biopsies cannot provide information on treatment efficacy in real-time, RNA-based biomarkers are being developed to address these issues. A liquid biopsy platform that enables non-invasive real-time detection of cancer biomarkers may significantly reduce the need for tissue biopsy. Advancements in liquid biopsies are a key objective of precision oncology, with the goal of improving the diagnosis and treatment of cancer^[21]^.

Tumour derived liquid biopsy analytes in the blood include circulating tumour cells (CTC), circulating tumour DNA (ctDNA), exRNA, exosomes, and EVs^[21]^. CTCs and ctDNA are the two analytes that have more reported utility than others as biomarkers in precision oncology. CTCs are tumour cells that have presumably been shed from the primary tumour and/or metastatic lesions into the bloodstream. CtDNA can be detected in the blood as part of the total cell-free DNA (cfDNA) pool, but is specifically derived from cancerous cells^[22]^. Clinical applications for CTCs and cfDNA include prediction of cancer prognosis, selection and monitoring of therapeutic regimens, and drug target applications^[21]^.

### Current challenges in cancer diagnostics using liquid biopsy

While liquid biopsies are increasingly being used for molecular diagnostics in oncology, challenges remain. One limitation in using CTCs for clinical applications is the scarcity of CTCs in the blood. The abundance of CTCs in the blood is low (approximately 1 cell per 1 × 10^9^ blood cells in patients with metastatic cancer), and only a limited number of CTCs can be isolated from a single blood sample^[23–25]^. Similarly, ctDNA concentration can vary from 0.01% to 90% of total cfDNA and, in general, the amount of ctDNA increases with tumour burden^[26,27]^. These extreme low concentrations can make detection and analysis challenging. While CTCs can be analysed at the DNA, RNA, and protein levels, and provide information on functional cellular characteristics, analyses of CTCs provide limited information on tumour heterogeneity^[28,29]^.

CtDNA provides a more comprehensive view of the tumour genome as it reflects DNA released from multiple tumour regions or different tumour foci to capture tumour heterogeneity^[30–32]^. However, due to the high fragmentation rate and low abundance of ctDNA, and high background levels of wild-type DNA in blood, the analysis is particularly challenging. Whole genome sequencing of cfDNA suggests both cfDNA and ctDNA are likely derived from apoptotic cells^[33]^. While CTCs are shed from a tumour once it reaches a certain stage in development and ctDNA is released from dying cells, exRNA secretion (biogenesis) is a normal cellular process. This makes exRNA and EVs better candidates to provide insight into early stage cancers where cell death is not yet occurring.

## LIQUID BIOPSY AND EXRNA

ExRNAs and EVs are among the liquid biopsy analytes that have demonstrated potential as cancer biomarkers due to their function, availability in most body fluids, and ability to be collected in a non-invasive manner, allowing frequent and longitudinal sampling. In cancer research, there is substantial evidence to support the use of exRNA for both diagnostic and prognostic purposes^[18]^. Differential expression of cellular and extracellular miRNAs has been associated with a wide range of human diseases^[34]^. While exRNA can include many diverse types of RNA species (as discussed above), most studies investigating the use of exRNAs as biomarkers have focused on miRNAs since its expression patterns are unique to individual tissues and differ between cancer and apparently uninvolved tissues^[35]^. Y RNA is abundantly expressed in multiple body fluids and increased levels of Y RNA have been observed in the circulation of cancer patients^[5,36]^. These observations have triggered interest in the potential use of Y RNA as a biomarker for cancer and other diseases. However, many other types for exRNAs are being explored as potential biomarkers, including mRNA mutations and other non-coding RNAs^[18]^.

There are opportunities for exRNA to be developed into reliable biomarker tests for cancer detection using liquid biopsy samples, since exRNA is remarkably stable and resists degradation mediated by ribonucleases^[15,37]^. The discovery of stable RNA or exRNA outside of cells is continuously changing the fundamental understanding of intercellular signalling and of the role RNA may play in cell-to-cell communication and other complex biological processes. Because of their relative stability within vesicles or in association with RNPs and LPPs, and, marked differences between exRNAs secreted by apparently normal and tumour cells, exRNA molecules have high potential for development as biomarkers of various cancers including lung^[38]^, breast^[39]^ and prostate cancers^[40,41]^.

## ADVANTAGES OF EXRNA IN LIQUID BIOPSY

The remarkable stability and relatively non-invasive access of different exRNA molecules makes them an interesting class of biomarkers. The stability of exRNAs have been tested*ex vivo* under various conditions including freeze-thaw cycles, extreme pH values, and storage at room temperature (RT)^[42]^. Examination of exRNA from CSF or blood in the diagnosis of glioma revealed that the EV number and morphology remained largely unchanged if CSF was stored at RT^[43]^. The total RNA and representative miRNA levels were well-preserved under this condition for up to a week, and a single cycle of freezing and thawing did not significantly alter EV number, morphology, RNA content, or miRNA levels, confirming its stability at RT. These findings demonstrated stability and the incredible ease and speed of obtaining specimens for testing compared to conventional biopsy. Measuring circulating RNA as liquid biopsy is a reliable alternative to conventional biopsies, offering a potentially cheaper, reliable, and non-invasive way of monitoring cancer development, progression, and remission.

Most of the tests for disease diagnosis and monitoring used in clinics are based on specific protein concentration changes in body fluids. In comparison to protein-based biomarkers, exRNA has several advantages including easier developed assays with specificity, and an amplifiable detection signal. Measuring low abundance RNA in biofluids also circumvents the inherent obstacle of high protein concentration and complexity in human body fluids in liquid biopsy.

In various diseases, normal EV cargo contents change as diseases initiate, and progress, altering the types of proteins and RNAs that are packaged. These changes are rapid and quantitative^[44]^. Therefore, the exRNA profile of an individual may provide a snapshot of their health. Real-time changes in expression of exRNA offer prognostic values in predicting disease outcomes, monitoring treatment response and assessing treatment risk^[45]^.

## EXRNA AS CLINACAL BIOMARKERS

In order to develop exRNAs as clinical biomarkers, the development process has to go through rigorous steps to define the intended target, examine clinical utility (must inform and guide patient treatment, management, and outcomes) and, validate the test both analytically (ensures specificity, accuracy, precision, and other characteristics of a biomarker test or device) and clinically (ensures that the test or device performs as intended) before clinical application. Since the U.S. Food and Drug Administration (FDA) is the regulatory body to qualify biomarkers for intended clinical studies, it is therefore relevant to understand the FDA definition of a biomarker and review various resources available for investigators.

### FDA’s definition of a biomarker

The FDA defines a biomarker as a defined characteristic that is measured as an indicator of normal biological processes, pathogenic processes, or responses to an exposure or intervention, including therapeutic interventions^[46,47]^. Qualified biomarkers have the potential to provide valuable information that may reduce uncertainty in regulatory decisions during drug development. When a biomarker is qualified, it means that it has undergone a formal regulatory process to ensure that it is reliable and reproducible for a specific interpretation and application in medical product development and regulatory review, within the stated context of use.

### FDA BEST biomarker categories resource

It is essential to have effective, unambiguous communication for efficient translation of promising scientific discoveries into approved medical products. Unclear definitions and inconsistent use of key terms can hinder the evaluation and interpretation of scientific evidence and may pose significant obstacles to medical product development programs.

The FDA-NIH Joint Leadership Council identified harmonization of terms used in translational science and medical product development as a priority need, with a focus on terms related to study endpoints and biomarkers. Working together with the goals of improving communication, aligning expectations, and improving scientific understanding, the FDA and NIH developed the BEST (Biomarkers, EndpointS, and other Tools) resource for biomarker researchers^[46]^. BEST defines seven biomarker categories: susceptibility/risk, diagnostic, monitoring, prognostic, predictive, pharmacodynamic/response, and safety. The BEST glossary aims to capture distinctions between biomarkers and clinical assessments and describes their distinct roles in biomedical research, clinical practice, and medical product development.

### FDA center for drug evaluation and research biomarker qualification program

The mission of this program is to work with external stakeholders to develop biomarkers as drug development tools. Qualified biomarkers have the potential to advance public health by encouraging efficiencies and innovation in drug development. The goals of the biomarker qualification program (BQP) are to (1) support outreach to stakeholders for the identification and development of new biomarkers; (2) provide a framework for the review of biomarkers for use in regulatory decision-making; and (3) qualify biomarkers for specific contexts of use that address specified drug development needs.

Biomarker qualification is a process involving three stages that provide increasing levels of detail for the development of a biomarker for its proposed context of use. The processes to complete submissions to the center for drug evaluation and research (CDER) BQP are (1) a letter of intent (LOI); (2) qualification plan; and (3) full qualification package. More information about the FDA CDER BQP can be found on their website^[47]^. A Pre-LOI meeting can be helpful for requesters to receive guidance from the FDA regarding their biomarker programs before submission to the program^[48]^. Once a biomarker is qualified it can then be used in multiple drug development programs for the context of use without FDA re-review.

### ExRNA as cancer biomarker

Medical oncologists have been using cancer biomarker tests to guide molecularly targeted therapies to achieve better therapeutic outcomes. In this regard, developing biomarkers and biomarker tests based on exRNA is relevant and important in the context of precision cancer therapy. Investigators have been assessing the current state-of-the-art methods for body fluid sample collection, exRNA isolation, and analysis, with exRNA biomarker discovery as the goal. This data has been unified in a report on the current state of knowledge of exRNA isolation and analysis techniques^[49]^. To avoid loss of potential biomarkers, investigators have been using comprehensive methods, such as qRT-PCR and cutting-edge platforms for RNA sequencing, rather than selection methods for specific RNA species.

ExRNAs have already begun to demonstrate their utility as clinical biomarkers. A study by McKiernan *et al.*^[50]^ reported the development of a urine exosome-based non-invasive gene expression assay that discriminates high-grade from low-grade prostate cancer and benign disease. In another study Li *et al.*^[51]^ identified and validated a panel of salivary exRNA biomarkers for potential use in screening and risk assessment for gastric cancer. Using salivary gland secretions, investigators have identified 30 mRNA and 15 miRNA candidates whose expression patterns were associated with the presence of gastric cancer^[51]^. These exRNA biomarkers were identified and validated with credible clinical performance for non-invasive detection of gastric cancer. Another recent study reported analysis of ctDNA and exRNA for monitoring tumour burden and therapeutic response in patients with multiple myeloma^[52]^. This exploratory analysis has provided evidence of ctDNA for predicting disease outcome and the utility of exRNA as a biomarker of therapeutic response in multiple myeloma. It has been reported that an exosome-based detection of EGFR T790M in plasma from non-small cell lung cancer patients (NSCLC) may benefit from ALK (anaplastic lymphoma kinase) inhibitor therapy whose tissue samples are not available or who are unable or unwilling to undergo repeat biopsy^[53]^. To address this need, Exosome Diagnostics developed an assay (ExoDx *Lung-ALK*) in a CLIA certified laboratory to isolate and analyse exosomal RNA from blood samples enabling sensitive, accurate and real-time detection of EML4-ALK mutations in patients with NSCLC.

Exosome Diagnostics has also developed a qPCR-based test (ExoDx *EGFR*) that interrogates mutations within the *EGFR* gene in NSCLC. The assay uses plasma derived exosomal RNA/DNA and cfDNA to detect *EGFR* mutations to inform clinical management^[53,54]^. Castellanos-Rizaldos and colleagues compared this assay to the FDA approved companion diagnostic, cobas® *EGFR* Mutation Test v2 (Roche), that detects defined mutations within the *EGFR* gene from plasma cfDNA liquid biopsy samples of NSCLC patients and found increased sensitivity and specificity using the ExoDx *EGFR* assay which they attributed to the exRNA-based assay design^[53]^.

## EXRNAS AS BIOMARKERS OF CLINICAL SIGNIFICANCE IN CANCER

**Figure 3.**
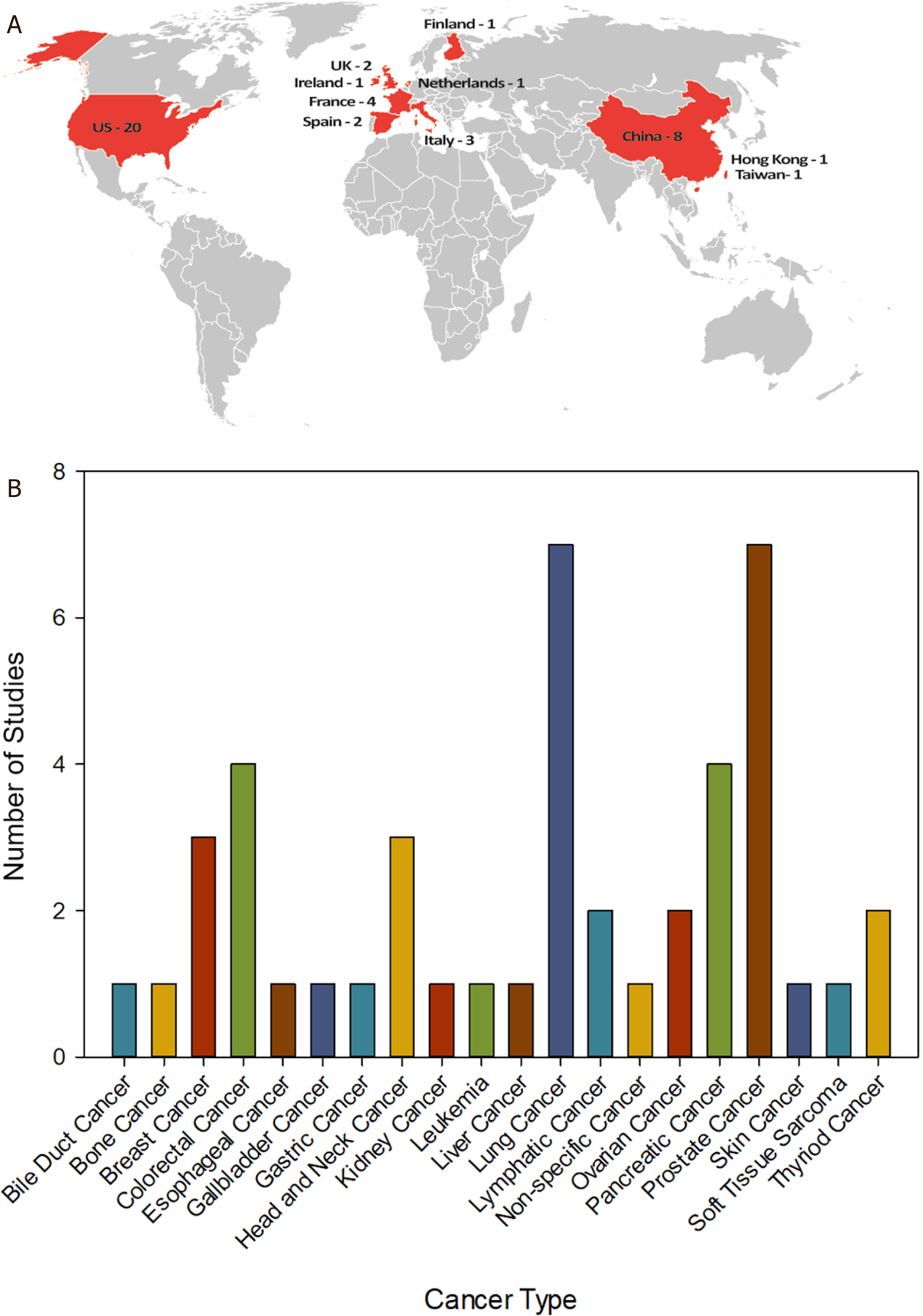

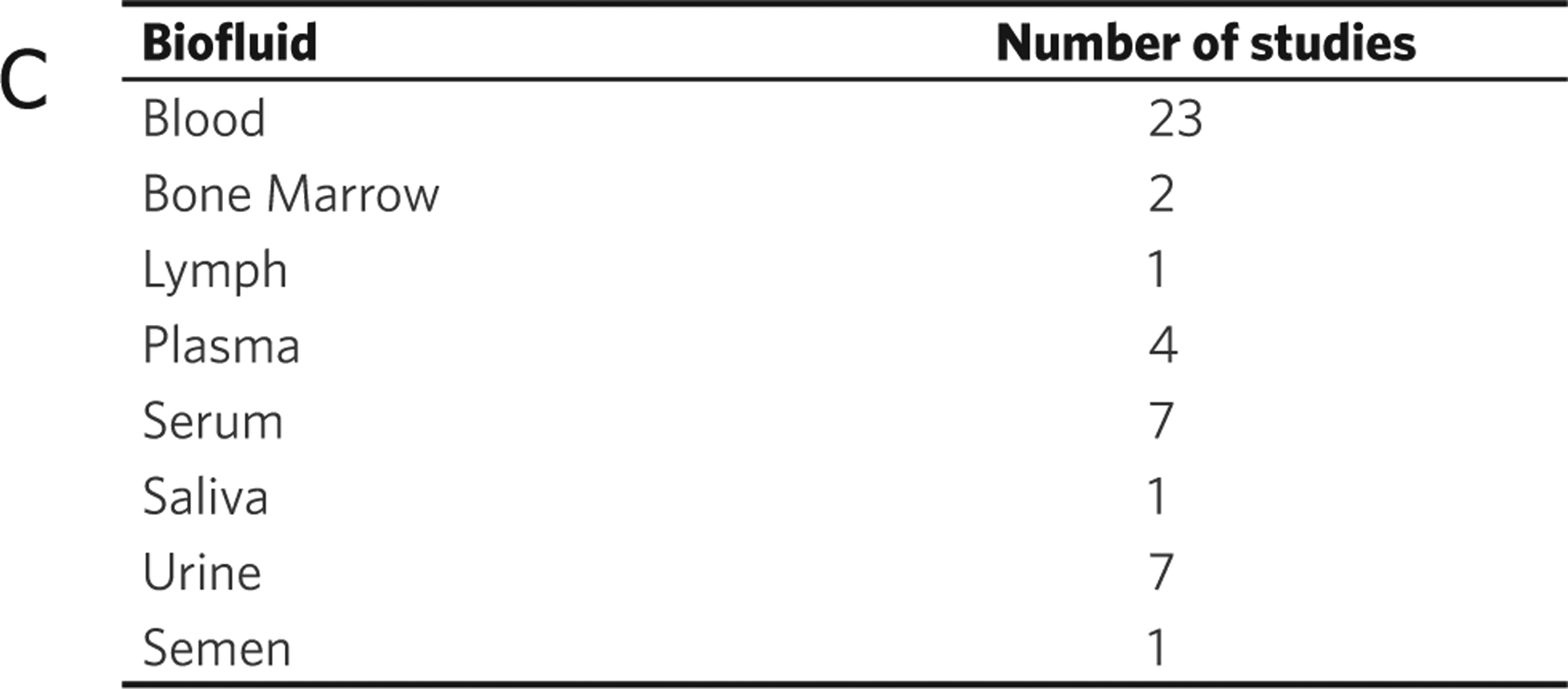
Current clinical studies evaluating the use of exRNA and exosomes as cancer biomarkers. An advanced search for query terms (“extracellular RNA” OR exosome OR exRNA OR oncosome) AND biomarker focused on cancer as a disease model, was performed on ClinicalTrials.gov on 11 May 2020. The search was restricted to recruitment statuses on recruiting, not yet recruiting, active, not recruiting, completed, enrolling by invitation, and studies of unknown status. The search returned 45 studies which are summarized in Figures 3A–C. A: A world map shows the locations (in red) of all clinical studies evaluating the use of exRNA and exosomes as cancer biomarkers. The numbers indicate the clinical studies in each location; B: clinical trials were grouped by general cancer type and the number of studies focused on each cancer type are shown. Projects that did not specify cancer type were grouped together as non-specific cancer; C: the clinical trial data was parsed for the types of biofluids used in each study. Some studies examined multiple types of biofluids while other did not include biofluid sampling. The table represents biofluids examined in all 45 clinical studies

To demonstrate the potential of exRNA and exosomes as clinical biomarkers, we mined data from current clinical trials exploring the utility of these liquid biopsy analytes in cancer. The ClinicalTrials.gov is a database for publicly and privately supported research studies conducted around the world. As of May 11th, 2020, there are 45 clinical trials on ClinicalTrials.gov that focus on the use of exRNA and exosomes as clinical biomarkers in cancer^[55]^. The search results are summarized in Figure 3A–C. While a vast majority of the clinical trials are taking place in the U.S., there are many trials in other countries as well, including China, Italy, and Spain [Figure 3A]. These clinical trials span a large variety of cancer types [Figure 3B]. Lung and Prostate cancers are the most common disease models exploring the use of exRNA and exosomes as clinical biomarkers, as both cancer types are the focus of seven clinical studies. Overall, there is a large number of different cancer types represented in this data set. Fourteen out of the twenty different cancer types are the focus of one or two clinical studies, demonstrating the utility of exRNA and exosomes as clinical biomarkers. Blood is the primary biofluid utilized in these studies while urine is also a common source of biofluid used for liquid biopsies [Figure 3C]. The combination of blood/serum/plasma is utilized for 34 out of the 45 clinical trials investigating the potential of exRNA and exosomes as clinical biomarkers, representing 17 different cancer types. Not surprisingly, urine is the biofluid of choice when investigating biomarkers for prostate cancer. However, clinical studies are also exploring the of use of urine as a biomarker for thyroid and kidney cancer. Saliva is an emerging biofluid that is inherently easy to collect, and, has been shown to reflect the spectrum of health and disease states found using serum^[56,57]^. While there is only one clinical trial in this dataset using saliva for biomarker discovery, it is conceivable that emerging technological advancements will move saliva into the forefront as an accurate and reliable biofluid for molecular diagnostics.

Most studies investigating the use of exRNAs as biomarkers have focused on miRNAs, and indeed 12 of the 45 clinical trials in this dataset specifically examine the use of miRNAs as cancer biomarkers. However, two studies investigate mRNA as exosomal cargo and a molecular biomarker in cancer. Further, there is a clinical study looking at circular RNA (cRNA) in pancreatic cancer, and a study exploring exosome derived lncRNA in ovarian cancer. Notably, most of the clinical studies did not indicate a specific exRNA target. Overall, this data demonstrates widespread utility of exRNA and exosomes as clinical biomarkers across a spectrum of biofluids and cancer types.

### NIH-supported research focused on exRNA and exosomes as biomarkers in cancer

**Figure 4.**
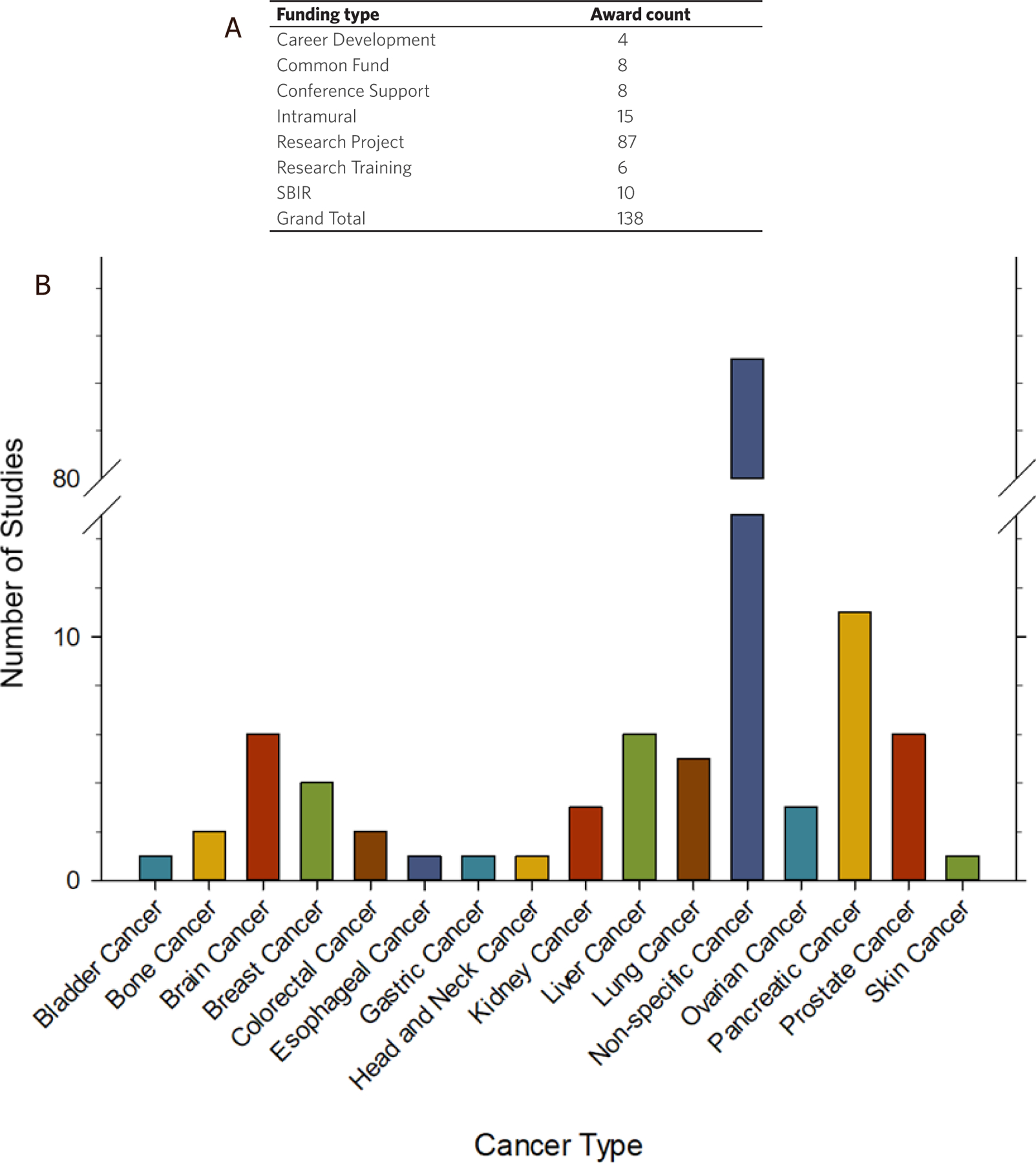
NIH supported research focused on exRNA and exosomes as cancer biomarkers. An advanced text search for (“extracellular RNA” OR exosome OR exRNA OR oncosome) AND cancer AND biomarker was performed on NIH RePORTER (https://projectreporter.nih.gov/reporter.cfm) on 9 May 2020. The text search was limited to project abstracts, project title, and project terms, and was focused on new awards only (excluding subprojects), funded by any NIH Institute or Center from 2010–2020. The search returned 138 projects, which are summarized in Figures 4A and B. A: NIH funded projects were grouped by general funding types; B: NIH funded projects were grouped by general cancer type and the number of projects focused on each cancer type are shown. Projects that did not specify cancer type were grouped together as non-specific cancer

The NIH supports many pre-clinical research projects focused on the use of exRNA and exosomes as biomarkers in cancer. To understand the breadth and type of research funded by NIH, we explored the Research Portfolio Online Reporting Tools Expenditures and Results Tool (RePORTER) using the website: https://projectreporter.nih.gov/reporter.cfm. An NIH RePORTER search for exRNA and exosomes as biomarkers in cancer found 138 projects that have been funded by NIH between 2010–2020, which is summarized in Figure 4A and B. NIH funded projects were grouped by funding type and the number of awards for each funding type can be found in Figure 4A. A large majority of these studies (87 out of 138) were research projects. However, the number of research training and career development awards indicate a growing number of trainees entering the field. The NIH Common Fund’s Extracellular RNA Communication Program (ERCP) funded eight projects focused on exRNA and exosomes as biomarkers in cancer. NIH Small Business Innovation Research (SBIR) awards make up 10 of the 138 projects. These SBIR awards included funding to Tymora Analytical Operations, Cognext Diagnostics, Abtelum Biomedical, Nanomaterial Innovation, Biofluidica, Nanoview Diagnostics, Accure Health, Ascent Bio-Nano Technologies and Microsensor Labs.

A large majority (85 out of 138) of the NIH funded projects that focused on exRNA and exosomes as biomarkers in cancer were not directed toward any specific cancer type [Figure 4B]. Furthermore, the 53 remaining projects were spread across 15 different types of cancer.

Pancreatic cancer was the focus of 11 studies over the past 10 years and accounted for 8% of the total number of awards. Brain, liver, and prostate cancer were each investigated in 6 studies, and the remaining cancer types were each addressed 5 or less times. This data, along with the clinical trial data, suggest that exRNA and exosomes have great potential as biomarkers in a variety of cancer types and across many types of biofluids. The broad applicability, universal presence in human biofluids, general stability, and accessibility of exRNAs demonstrate their potential in disease detection, monitoring, and prognosis.

### FDA-approved exosome-based clinical diagnostics

Exosome Diagnostics (a Bio-Techne brand) recognized an opportunity to utilize exRNA as a predictive marker for prostate cancer and developed a urine exosome gene expression assay that can identify higher-grade prostate cancer among patients with elevated prostate-specific antigen (PSA) levels. This simple, non-invasive, urine-based test provides an EXO106 score derived from exosome ERG and PCA RNA levels normalized to SPEDEF mRNA copy number^[50,58]^. The U.S. FDA granted Bio-Techne Breakthrough Device Designation to this test [ExoDx Prostate IntelliScore (EPI)], making it the first exosome-based liquid biopsy test to receive this designation, and Medicare coverage in 2019. Further, a recent publication demonstrated that the EPI test influenced the overall decision to defer or proceed with a biopsy and improved patient stratification in a prospective, randomized, blinded, two-armed clinical utility study^[59]^.

## CHALLENGES IN EXRNA RESEARCH

Even though the field of exRNA is very promising, there are challenges to this emerging area. A key barrier toward a comprehensive understanding of exRNA biology and function has been the heterogeneity of exRNA carriers, improved EV separation technologies, and EV targeting and cargo release.

### EV biogenesis and cargo loading

ExRNA carriers include different particle subtypes such as EVs, RNPs, and LPPs, however, EVs have gained the most interest amongst these carriers. EVs are highly heterogeneous and can be further divided into different subpopulations that differ in size, density, morphology, and composition^[60]^. EV subpopulations broadly include MVs and exosomes^[60]^. An ongoing challenge in the field is to clearly discriminate between EVs, exosomes, and MVs.

Different EV biogenesis pathways also result in exRNA content that is extremely diverse and heterogeneous; and the intracellular sorting mechanisms that direct exRNAs to specific export pathways are not well understood^[61,62]^. Furthermore, the nature and abundance of EV cargoes are cell-type-specific and often influenced by the physiological or pathological state of the donor cell and the stimuli that modulate their production^[63]^. EV heterogeneity and the complexity of its exRNA cargo are likely sources of variability in exRNA profiling. Understanding the molecular mechanisms modulating EV biogenesis, the heterogeneity in EV subtypes, and the physiological relevance of their exRNA cargo will be crucial in harnessing their utility as cancer biomarkers.

### Single vesicle EV isolation

A major challenge to the field of exRNA includes improved EV separation technologies. The heterogeneity of EVs, their nanoscale size, and the ambiguity of EV subpopulations that often have overlapping characteristics, are significant barriers to understanding the contribution of each specific EV subtype in different pathological systems^[60]^. Due to a substantial overlap in the physio-chemical properties of exRNA carriers, many commonly used isolation protocols do not unambiguously separate EVs subtypes, or even EVs from non-EV exRNA carriers (such as RNPs or LPPs)^[64]^. The lack of biophysical and biochemical markers for many different exRNA carriers makes the analysis and interpretation of exRNA data uniquely challenging. To address the variability in exRNA profiling studies, Murillo and colleagues applied computational deconvolution to exRNA-seq and exRNA qPCR profiles found in the Extracellular RNA Atlas (https://exrna-atlas.org). Their analysis led to the identification of six exRNA cargo types found in multiple biofluids^[65]^. While their findings suggest associations of cargo types with distinct carriers, it also demonstrated that the heterogeneity of exRNA carriers and cargo types exceeds the capabilities of current experimental methods to isolate and investigate specific carrier subpopulations and their cargo in a reproducible way^[65]^. The generation and optimization of methods to isolate high purity exRNA subpopulations from biological samples, and, analyse the subsequent carrier exRNA contents, is a current goal in the field.

### EV targeting and cargo release

To be functional in the context of cell-cell signalling, an EV must also be able to find its physiological target and release its cargo. But the question of how EVs target recipient cells can elicit a functional cellular response is still unknown. The specificity of targeting EVs to recipient cells is thought to occur through specific ligand-receptor interactions resulting in EV uptake. Mediators of these interactions include tetraspanins, integrins, lipids, lectins, heparan sulphate proteoglycans, and extracellular matrix components^[60,66]^. Once EVs are bound to the recipient cells, many different types of endocytotic processes are known to mediate cellular uptake^[13,60]^. Membrane fusion is an alternative entry method in cancer cells^[67]^. However, different mechanisms of internalization have been described for different cell types, and the mode of EV entry into target cells is thought to play a role in the functional effects^[66]^. It is possible that a population of EVs can simultaneously trigger a number of different methods of entry into a cell, with the primary entry points depending on the cell type and EV cargo^[66]^. Understanding the mechanism of EV targeting and cargo release, and how this affects the functional fate of exRNA in recipient cells are outstanding questions in exRNA biology.

## ERCP

The NIH Common Fund-supported Extracellular RNA Communication Program (ERCP) was launched in 2013 to accelerate progress in this new area of biomedical research. The overarching goal of the ERCP has been to accelerate progress in the field exRNA biology and establish exRNA, and their carriers, as mediators of intercellular communication. The first phase (stage 1) of the NIH Common Fund-supported Extracellular RNA Communication Consortium (ERCC1) addressed five major challenges in the exRNA field^[68]^. The goals included: (1) to better understand the mechanisms of exRNA biogenesis, export and secretion from the cell of origin; (2) to develop reference profiles for exRNA species from healthy human biofluids; (3) to establish the utility of exRNA for biomarker development; (4) to establish the utility of exRNA for therapeutic development; and (5) to develop community-wide resources for exRNA standards, protocols, and data. The exRNA Portal (https://exrna.org/) is the central access point for ERCC resources including descriptions of all ERCC projects, exRNA data and data standards, protocols, and computational tools.

While significant advances were made during ERCC Stage 1, the exRNA field still faces many challenges, in part due to both the inherent diversity of exRNA and the heterogeneity of exRNA carriers^[61]^. In September 2019, the ExRNA Communication Program stage 2 (ERCC2) commenced to tackle the complexity of exRNA molecules and the diverse array of exRNA carriers. ERCC2 researchers will develop tools to efficiently and reproducibly isolate, identify, and analyse different carrier types and their exRNA cargos and allow analysis of one carrier and its cargo at a time. The three major initiatives addressed in Stage 2 of the ERCC include: (1) Improved Isolation and Analysis of exRNA-Carrier Subclasses; (2) Towards Single Extracellular Vesicle (EV) Sorting, Isolation, and Analysis of Cargo; and (3) to serve as a community-wide resource for exRNA standards, protocols, and data. The purpose of these initiatives is to further characterize the cell or tissue from which their respective exRNAs originate and shed light on the diversity of exRNAs carried by EVs. This will allow for a better understanding of the precise role of exRNAs as signalling molecules for both physiological and pathophysiological processes, ultimately accelerating the development of exRNAs for diagnostics.

## CHALLENGES OF EXRNA IN LIQUID BIOPSY

Although exRNAs are more sensitive and specific biomarkers than proteins, and better reflect the cell dynamic than DNA does, there are limitations in the use of exRNA as biomarkers. EV heterogeneity and the complexity of its exRNA cargo, are sources of variability in exRNA profiling within and across studies, which has been a significant hinderance. To address the lack of consistency and reproducibility, Srinivasan *et al.*^[69]^ demonstrated that exRNA sequencing reproducibility varies across isolation methods and that the performance of exRNA isolation methods can vary across biofluids and RNA species. To stimulate exRNA biomarker development, they developed miRDaR (https://exrna.shinyapps.io/mirdar/), an interactive web-based application to help investigators select the optimal exRNA isolation method for their studies based on the biofluid of interest. The development of standardized sample isolation and analysis procedures would allow a more meaningful comparison and integration of data from different studies, which may facilitate the development of exRNA based clinical applications.

EVs are heterogeneous in nature and technical challenges remain in EV isolation. Current methods for isolating EVs from complex biofluids cannot clearly identify EV cellular origins within a pool of highly abundant vesicles. As such, there is no way to clearly differentiate cancer-derived EVs from healthy host cell-derived EVs in biofluids. However, ERCC2 efforts should be able to address this pressing challenge. A recent report described a process for EV enrichment by identifying cancer cell membrane proteins compared with healthy cell membrane proteins using TCGA Human Protein Atlas and GTEx, and presented isolation of tumour derived EVs from animal serum^[70]^. This finding is encouraging to pursue exRNA biomarker research for detecting cancer at a very early stage. Better characterization of the differences between exRNA profiles of diseased and healthy individuals will allow the diagnostic and prognostic utility of exRNA-based profiling to increasingly becoming a reality^[18,50,71]^.

## CURRENT STATUS OF EXRNA AS BIOMARKER

It is conceivable that EVs, exosomes, and exRNA are important resources for developing cancer biomarkers. In this regard, a growing number of scientific reports suggest exRNA as a reliable non-invasive alternative to the invasive approaches for diagnosis, treatment and prognosis of cancer. Recently, a U.S.A.-based diagnostics company utilized exRNA as a predictive marker for prostate cancer, and developed a urine exosome gene expression assay to identify higher-grade prostate cancer among patients with elevated PSA levels^[50,58]^. U.S. FDA granted Bio-Techne Breakthrough Device Designation to this test (ExoDx Prostate IntelliScore, EPI), which is the first exosome-based liquid biopsy test to receive this Designation. The National Comprehensive Cancer Center Network included EPI as a recommended test in their Clinical Practice Guidelines for Oncology for Prostate Cancer Early Detection (Version 1.2019). While this is a significant step forward in exosome/exRNA-based test development, advancement in this technology is required to address all types of cancers.

The explosion of technological advancements including sophisticated bioinformatics and availability of better tools offer a wide spectrum of opportunities to explore exosomes/exRNA for developing reliable biomarker tests using liquid biopsy samples to accelerate real-time cancer diagnosis and molecularly guided therapy.

However, there are challenges to isolate tumour-specific exRNA and use as biomarkers for clinical oncology due to inadequate separation technology and heterogeneity of exRNA carriers. Current methods for isolating EVs from complex biofluids does not clearly define the cell-of-origin or target cell of exRNA cargo and, therefore, are unable to determine with certainty the tissue of origin. This warrants improvement in EV separation technology, and better understanding of EV targeting and cargo release.

The expectation is to develop liquid biopsy-based analytical assays using circulating exRNAs specific for the tumour type and to identify clinically relevant biomarkers useful as a diagnostic, prognostic or treatment response markers for cancer patients to fully appreciate its clinical potential as cancer biomarkers.

## FUTURE PERSPECTIVES

The potential for the use of EVs, exosomes, and exRNAs in cancer biomarker development are starting to yield clinical utilities for diagnosing cancer, and as indicators of progression and/or treatment response. EVs derived from cancer cells appear to modulate the function and may induce epigenetic changes in distant recipient cells. Results from several studies as indicated in this review have already shown a prominent role of exRNAs associated with exosomes in instituting these changes. EVs can retain the molecular signature of the cell of origin, and its exRNA cargo has tremendous diagnostic potential. Since the identification of exRNAs in various human bio-fluids, an increasing number of studies have positioned exRNA as a new type of non-invasive biomarker with wide-ranging clinical potential.

While significant advances have been made, the use of exosomes and exRNAs as cancer biomarkers faces remaining challenges that slows down its full potential from being realized. The NIH-led ERCC has supported research into the important roles of exRNAs in biological processes and its potential in molecular diagnosis, and to advance the technologies of exRNA identification and isolation from different types of bio-fluid. The ERCC has played critical roles in unmasking the mechanism of exRNA biogenesis, delivery and function; in defining a reference catalogue of exRNA in normal individual body fluids; in developing the clinical utility of exRNA as biomarkers of disease or as therapeutic molecules. The ERCC have also led the field in addressing major challenges in the field and providing valuable tools and technologies in this emerging field.

Although a few exRNA biomarkers have been discovered individually for cancer diagnosis, a systematic identification of novel exRNA biomarkers will need to be further pursued through better isolation of homogeneous populations of exosomes and comprehensive analyses of their cargo. Currently, there are only limited mature exRNA biomarkers that could guide clinical decision making. Large cohorts with matched clinical information, including survival time, disease recurrence, response for drug usage or other information can be catalytic in the identification of novel exRNA biomarkers. Sufficient clinical cohorts are also required to validate the performance of biomarkers for early-diagnosis, prognosis and drug usage for precision oncology.

In the future, it is also possible to target exRNAs as cancer therapeutic methods. The secretion and circulation of EVs that contain regulatory exRNAs can be blocked to prevent cancer from progressing and metastasis developing. In addition, exosomes could be used as a transmitter of specific regulatory elements into target cells, inhibiting the development of tumour. Some regulatory exRNAs that play roles in pivotal processes in tumour development could be repressed or sequestered to lower their abundance and inhibit their functions. In summary, exRNA is useful not only for liquid biopsies to diagnose various cancer types, but it also provides potential avenues for therapy.

## Figures and Tables

**Table 1.** General classification of non-coding RNAs

Group	Abbreviation	Full Name	Size	ncRNA Review Article(s)
Housekeeping ncRNAs				
	rRNA	ribosomal RNA	120–4,500 nt	[72,73]
	snRNA	small nuclear RNA	100–300 nt	[74]
	snoRNA*	small nucleolar RNA*	60–300 nt*	[75,76]*
	tRNA	transfer RNA	76–90 nt	[77]
Regulatory ncRNAs				
	lncRNA	long non-coding RNA	> 200 nt	[78]
	miRNA*	microRNA*	21–22 nt*	[79,80]*
	piRNA*	piwi-interacting RNA*	23–31 nt*	[81]*
	siRNA	small interfering RNA	20–25 nt	[82]
	tRF*	tRNA-derived fragments*	17–26 nt*	[83]*
	Y RNA*	Y RNA*	?*	[84]*

* Asterisks represent the most prominent types of exRNAs found in human biofluids
